# Attitude and practices of district-level HMIS managers towards malaria routine reporting in Uganda: a 2024 cross-sectional study

**DOI:** 10.1186/s12936-026-05951-8

**Published:** 2026-06-01

**Authors:** Simon P. Kigozi, John Okiring, Lameca Ssebagala Kigozi, Paul Emuron, Ephraim Alikosi Talimusa, Ruth N. Kigozi, Victor Alegana, Benn Sartorius, Emanuele Giorgi, Chris Drakeley, Adoke Yeka

**Affiliations:** 1 Department of Disease Control & Environmental Health, Makerere University School of Public Health, Kampala, Uganda; 2 Department of Disease Control, London School of Hygiene & Tropical Medicine, London, UK; 3 Malaria Consortium, Kampala, Uganda; 4 WHO Regional Office for Africa, Brazzaville, Congo; 5 UQ Centre for Clinical Research (UQCCR), Faculty of Medicine, University of Queensland, Brisbane, Australia; 6 Centre for Health Informatics, Computing and Statistics (CHICAS), Lancaster University, Lancaster, UK; 7 Department of Infection Biology, London School of Hygiene & Tropical Medicine, London, UK

**Keywords:** Surveillance, Malaria, Routine reporting, HMIS, Attitude, Practices, Data quality, Support supervision, District

## Abstract

**Background:**

Routine surveillance through the health management information system (HMIS), has taken a de facto management structure centered on district leadership, aided by the district health information software for reporting. System performance and credibility has long been derived on the basis of data indicators with little to no consideration of dimensions on human-resources in charge. This study therefore aimed to assess attitudes and practices of HMIS managers at district-level.

**Methods:**

A cross-sectional study was conducted across all 15 malaria endemicity regions of Uganda between January and June 2024. Semi-structured interviews were conducted among HMIS managers in at least one district-level health office per region. The primary outcomes of the study included attitude and practices in malaria routine reporting, particularly data recording, review, reporting, analysis, and use, as well as support supervision. Results were summarized using descriptive statistics and word clouds.

**Results:**

The 34 participants from 30 districts and cities included biostatisticians (70.6%) and HMIS focal persons (23.5%), overseeing 6 to 1043 actively reporting health facilities. All participants reviewed the reports they received, with 75.8% reporting documenting the mistakes found, though only 31.6% could show their documented queries. By survey date, 81.6% of expected routine reports had been received by the district-level health office, with 25.4% of them received after active follow-up with health facilities. With nearly all data submitted to DHIS-2 by the 15th day of the new month, 93.9% received queries constituting a mean of 4.2 queries per implicated health facility. Whereas ≈70% preferred quarterly DHI support supervision visits, 39.4% had received one to two such visits while 51.5% provided support supervision to their facilities, over the past 12 months. Generating mostly summary tables, trend and endemic channel plots, key data uses included performance reviews, resource (re)allocation, as well as staffing needs’ assessments.

**Conclusion:**

Highly capable human resource teams at district-level health offices administered HMIS routine reporting. Teamwork with health facility officials provides a framework for HMIS strengthening. Gaps remain in: use of or adherence to standard operating procedures for data reporting, feedback, and follow-up; conducting scheduled and/or evidence-driven support supervision; and, confidence in and advanced analytical skills facilitating improved data use.

**Supplementary Information:**

The online version contains supplementary material available at 10.1186/s12936-026-05951-8.

## Introduction

Malaria control and elimination heavily rely on the vigilance of health systems’ human resources to adequately and ardently perform routine surveillance activities, both passive and active [1]. In sub-Saharan Africa, routine surveillance of all reported diseases has adopted a de facto management structure, hinged around district-level headship, through which the mainstay reporting system, introduced around 2010, derives the name District Health Management Information Software—Version 2 (DHIS2) [2–4]. As such, duty performance of health management information systems (HMIS) surveillance activities by district or city health management officials (DHM), is critical. In high endemicity settings, malaria surveillance activities at the district involve the collation of data from district-supervised health facilities, monitoring quality of these data, and submitting the abstracted data to the national central authorities—mainly National Malaria control divisions/Programs (NMCP) under the leadership of national ministries of health.

The Ugandan health system is comprised of a top-down hierarchy of health facilities including national referral, regional referral, and general hospitals; health centres IV, III, and II; and village health teams. It follows a hierarchy in routine reporting too, using a standard report form, HMIS 105, predominantly submitted in physical form from lower-level facilities to the respective health sub-district or district health office each month. Timely submission of a monthly report is achieved if it’s received by DHM no later than the seventh day of the next month [5]. Higher level hospitals have largely had direct access to the DHIS2 reporting system, thereby submitting reports online. As one goes up the reporting levels, data is submitted in aggregate format, but it remains identifiable by its source facility, sub-county, and district. Among a plethora of standard tools is Annex 8G for DHM tracking of receipt, validity, and completeness of reports per facility [6]. At DHM, monthly reports should be validated on a quarterly basis and evidence of action taken on findings kept [7]. When analysed, outputs from HMIS are expected to be discussed during quarterly performance review meetings, among others. However, the level of adherence to these standards in practice remains unclear.

Enormous investments have been made to strengthen HMIS, through the development of increasingly complex data collection tools and derivative indicators and the establishment of electronic reporting systems, for example [8, 9]. Despite recent improvements, some inconsistencies still exist in reporting, as well as data errors and timeliness challenges, among others [10, 11]. Notably however, perspectives and practices of frontline HMIS data handlers in district-level health offices, who play a data gatekeeper role between health facilities and national health authorities, have hardly been assessed. Improved understanding of these aspects at this management level may foster adapted surveillance support systems and highlight key target points in the pathway to surveillance transformation into a distinct intervention. This study therefore, aimed to assess the attitude and practices of frontline officials in HMIS data recording, reporting, analysis and use, and associated support supervision, at district level.

Routine HMIS data plays a central role in surveillance-based evidence generation and health facility monitoring, given the mandate to analyse and use it [1, 7, 12]. Efforts towards its improved interpretation and thereby use include integration of HMIS with supportive data from other sources [13, 14]. Notably for Uganda, the prevailing malaria reduction and elimination strategic plan 2021–2025 set as its fourth of six strategic objectives, to have “malaria programming at all levels guided by and based on robust data and evidence” [15]. Despite all efforts of good intention to improve HMIS data, generate evidence through advanced analyses, or caution about the data itself, ignoring the role of HMIS data handlers may perpetuate limitations to its full utility. Moreover, majority of factors associated with improved HMIS data use, including prioritization of data or promotion of a data-use culture, skills training, support supervision, regular performance review, planning, governance, and resource availability, are all people-centered [1, 16–18]. Extensive progress has been made in and attention placed on advancing modeling approaches to better interpret HMIS data with commendable benefits [19–22]. However, the potential role of top-level managers in the state of routine HMIS data, considering their perceptions and day-to-day actions, remains little understood or appreciated. An improved understanding of this perspective could facilitate the identification of capacity needs and the establishment of accountability mechanisms, as well as, improved data ownership, interpretation, and use [23].

A formative cross-sectional assessment of attitudes and practices in management of routine data, among the DHM HMIS managers, presented a great opportunity to examine processes undertaken and unearth potential pitfalls and/or areas for improvement and intervention.

## Methods

### Study site

**Fig. 1:**
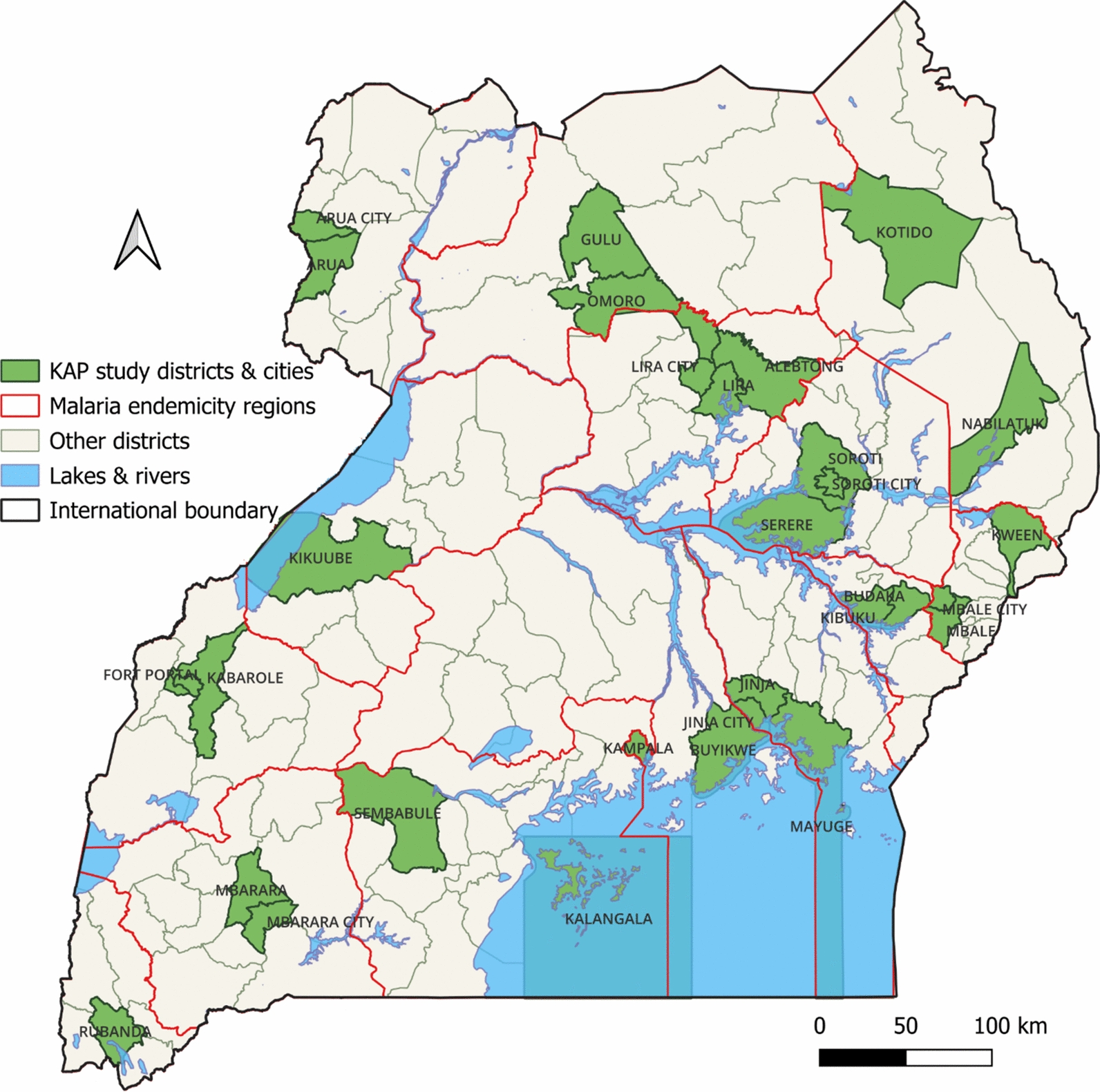
Study sites of districts and cities across Uganda, enrolled into the survey - map generated using Q-GIS 3.36.0 software

The study was conducted across 22 districts and 8 cities around Uganda (Fig. 1), ensuring at least one districts and/or city, was selected from each of the 15 malaria endemicity regions of the country, as established in 2018 [24]. Prior to the selection of study districts, all districts were classified as either predominantly rural or urban, based on UBOS 2023 report on district population distribution (urban versus rural). From each region, a random selection of a district, in each classification, was conducted by an independent statistician.

Interviews were conducted at the respective DHM offices, where routine HMIS reporting from respective health facilities and to the DHIS-2 is managed. The DHIS-2 is administered by the Division of health information (DHI), under the Uganda Ministry of Health.

### Study design

The study involved a cross-sectional survey, conducted between January and June, 2024, using a semi-structured interviewer-administered questionnaire.

### Study population and sampling

DHM directly involved in routine HMIS reporting were the study population from among whom, one participant per jurisdiction was approached, recruited and interviewed. All district/city health team members involved in HMIS reporting were eligible for recruitment into the study, if agreeable to consent. DHM offices were visited by a research assistant and from each office, one member of the DHM team that is actively engaged in HMIS reporting was recruited into the study, except Kampala city. Kampala health management is further decentralized into five divisions. Here, one member of each division’s health team, actively engaged in HMIS reporting, was recruited into the study. Particular interest was placed on biostatisticians, HMIS focal persons, and district-level health officers, but in the absence of all these, a designate handling HMIS data for routine reporting purposes was considered.

### Data collection

A semi-structured, study validated questionnaire was developed and implemented on the Kobo Toolbox platform [25] to be administered by trained study team members to consenting participants. The questionnaire was designed in English with both closed and a few open-ended questions categorized into the four topical sections of: (a) Data recording and review, (b) Data reporting (c) Support supervision, and (d) Data analysis and use. In each of these sections were questions addressed to attitude and practices. Participant demographics, including age, cadre, gender, and level of education were also captured at the start of the interviews.

Field team members were trained on the study protocol, the informed consent process, and administering of the questionnaire. Questionnaires were conducted in electronic format by the trained field workers, using android tablets with data regularly uploaded to a remote Kobo server. All interviews were conducted in English, with no need for translation, since all participants were expected to be sufficiently fluent in English.

District copies of the most recent monthly and support supervision reports, as well as documented data queries generated from review of received data were also sought and reviewed by the study team. Queries were defined, in this study, as inquiry statements or questions pointing to concerns around validity, accuracy, consistency, or missingness of content in a reviewed report that was considered complete at its source. Particular focus was paid to identify a sample of health facilities (one per level, including health centres II, III, IV, and hospital, as well as a private-for-profit (PFP facility) per district/city in these documents. Support supervision was assessed in two directions, first between DHI and districts and second between districts and their associated health facilities.

### Outcome variables

The primary outcomes included malaria routine reporting attitude and practices among district-level HMIS managers, handling routine/monthly reports that are regularly compiled and submitted to the district by health facilities.

To assess attitude, questions addressing considerations made prior to key actions, desired frequency of key actions, desired formats of action outputs, and perceptions of HMIS in decision-making, were addressed.

To assess practices, questions addressing actions and time to actions, such as: quality assessment, follow-up on quality concerns, and process tracking were used.

### Data analysis

Data was downloaded from the Kobo Collect server in comma separated version (CSV) file format and then transferred to STATA 18.5 (College Station, Texas 77,845 USA) for analysis. Data on all outcomes and other metrics of interest was summarized using descriptive statistics of responses on the survey questions addressed across each topical section. Open ended question responses were summarized into word clouds to estimate density of key terms, using the open access word-cloud generator platform on www.wordclouds.com, and therewith identify apparently outstanding sub-themes.

## Results

### Participant socio-demographic characteristics

The study recruited 34 participants from 30 districts and cities across Uganda, which was 80% of targeted sites (Fig. 1). 58.8% of participants were male and by age, majority were between 30 and 45 years, among both the male (60.0%) and female (71.4%) (Table 1). Participants predominantly held either the district biostatistician (70.6%) or HMIS focal person (23.5%) position, both being frontline management positions for HMIS reporting. Each staff team to which participants belonged, oversaw anywhere between 6 to 1043 actively reporting health facilities, and 70.6% of study participants had attained graduate or post-graduate level education. No significant associations were found between participants demographic characteristics including education and age, district status, or participant designation within the DHM, and any of the four outcomes pertaining to attitudes or practices in routine reporting (Additional file). Notably, however, district status (urban versus rural) and age (older age) showed identifiable patterns, worthy of further investigation, consistent in crude and adjusted regression models.

**Table 1:** District HMIS manager attitude and practices survey participants’ demographics summary

Variable	n (%) N = 34
Gender	
Male	20 (58.8)
Female	14 (41.2)
Age	
< = 30 years	7 (20.6)
31–45 years	22 (64.7)
46–60 years	5 (14.7)
Highest level of education	
Certificate	1 (2.9)
Diploma	9 (26.5)
Degree	11 (32.4)
Post-graduate	13 (38.2)
Participant Cadre	
HMIS focal person	8 (23.5)
Biostatistician	24 (70.6)
Other	2 (5.9)
Number of health facilities reporting on malaria to the district office – *(min, median, max)*	
Minimum	6
Median	28
Maximum	1043

### Data recording and review

Every participating district-level site had between one and eight (except one Kampala city division with 32) members of staff involved in handling routine reports being submitted to the district, by their health facilities. Majority of the districts (73.5%) used an electronic spreadsheet to monitor compliance of their health facilities at submitting monthly reports (Table 2). Whereas some health facilities used paper spreadsheets/registers or monitored through DHIS-2 dashboards, at least one third (33.3%) used more than one means of monitoring submission compliance by their health facilities, including one that preferred to simply wait for the facilities to submit.

**Table 2:** Attitude and practices towards routine data recording and review, among district HMIS managers

Question	n (%) N = 34
How do you keep track of facilities that have/have not submitted a monthly report? ^β^	
Use electronic spreadsheet	25 (73.5)
Use paper spreadsheet / register	11 (32.4)
Monitor on DHIS-2 or mTrac	7 (20.6)
When do you consider a monthly report delayed? ^@^	
> 7 days into the new month	23 (67.6)
2–10 days into the new month	6 (17.7)
> = 11 days into the new month	5 (14.7)
When do you get in touch with health facility if monthly report is delayed? ^β^	
5–10 days into the new month	21 (61.8)
10–15 days into the new month	12 (35.3)
> 15days into the new month	1 (2.9)
Do you document mistakes found in monthly reports you receive? ^β^	
Yes	26 (76.5)
No	8 (23.5)
How do you communicate mistakes identified in monthly reports to health facility? ^β^	
Phone call	30 (88.2)
SMS/WhatsApp message	16 (47.1)
Send documented query on paper	3 (8.8)

@ – Attitude/perception questions, β – Practice questions

For both keeping track of facilities submitting reports and communicating mistakes identified in the reports, some participants used more than one option

Whilst all participants reported that they always reviewed the reports that they received from health facilities, for completeness, accuracy and consistency, 23.5% indicated that they do not document the mistakes identified in the reports received (Table 2). Among those that did not document mistakes found, 62.5% (5/8) opted for calling the health facilities, by phone, to get the issue resolved as their preferred action. One participant particularly specified that they don’t proceed till the issue was resolved. Among mistakes’ non-documenters, taking a mental note, commenting directly on the report, and making a phone call to resolve issues were their primary approaches to keeping track of the mistakes they identified.

Following the identification of mistakes in reports submitted to the districts by health facilities, the majority of participants, 88.2%, indicated use of phone calls to the health facility staff to communicate the mistake, as well as get the mistake resolved (Table 2). Whilst majority of district officials use more than one means of communicating these identified queries in the health facility submitted monthly reports, phone calls form the majority when a single means of communication was used. Notably however, two participants indicated sending documented queries on paper to the facility staff, for correction in a single mode of communicating the mistakes identified. Notably also, none of the participants indicated sending the report back to the health facilities as an option of trying to resolve mistakes in reporting.

From a review of documented queries, among districts that reported having documented queries raised following their own review of the monthly reports for the most recent month, only 31.6% participants (6/19) were in position to provide a record of their documented queries. The others participants, despite reporting that they documented these queries, cited reasons such as: having communicated the queries to the health facilities and discarded their records; having recorded the queries on the health facilities’ copy of the submitted monthly reports, which are retained by facilities; and, inaccessible computer(s) on which the queries were recorded or unavailability of the documentation at the time, among others. Nevertheless, from the observed queries records, 91.7% of the selected health facilities (11/12) had at least one query to address.

Study assessment of the observed monthly reports received from sampled health facilities per district (at most 5 per district), showed that 81.6% of the expected monthly reports, relative to the date of the survey, had been received. However, of the reports received, 25.4% had been received after follow-up of the health facilities by the district-level health office.

Where report submission was delayed, majority of the districts (61.8%) got in touch with their associated health facilities within the first 5 to 10 days into the new month, with most of the rest not exceeding 15 days before they followed up. Regarding the duration when, after the reporting month, monthly reports were considered or perceived to be delayed, majority of participants (67.6%) indicated after seven days into the new month, while 17.7% considered a range of between two to ten days into the new month, as delayed.

### Data reporting

District reporting, involving entry of monthly report data into DHIS-2, was such that majority of districts (60.6%) entered their data by the 15th day, followed by within the first week of the new month (18.2%) (Table 3). The rest indicated doing entry as soon as reports were received or daily, at the district, or that entry was done by the health facility staff at the respective facilities.

**Table 3:** Attitude and practices towards routine data reporting, among district HMIS managers

Question	n (%) N = 34
When do you enter the reports data into DHIS-2? ^β^	
Daily	3 (8.8)
In the first week of the new month	6 (17.6)
By the 15th day of the new month	20 (58.8)
Other	5 (14.7)
How do you keep track of reports that you have submitted in DHIS-2? ^β^	
Counter sign on reports	19 (55.9)
Keep a data entry ledger	13 (38.2)
Rely on DHIS-2 dashboards	7 (20.6)
Not keeping track	1 (2.9)
Do you receive queries from DHI concerning what you submitted? ^β^	
Yes	32 (94.1)
No	2 (5.9)
How are data queries from DHI communicated to you? ^β^	
Email	18 (52.9)
SMS/WhatsApp electronic message	14 (41.2)
Phone call	8 (23.5)
Documented on paper	3 (8.8)
How do you keep track of the queries received from DHI? ^β^	
Refer to SMS/WhatsApp message received	19 (55.9)
Refer to email received	9 (26.5)
Refer to documented query on paper	7 (20.6)
Refer to my written summary from phone conversation	5 (14.7)
other	5 (14.7)
Was documentation of DHI-sourced queries availed to study staff for observation? ^β^	
Yes	14 (41.2)
No	20 (58.8)
Summary of observed DHI-sourced queries documentation	
Number of queries documented over 3 months	1201
Health facilities implicated in queries recorded over 3 months	283
Reasons for not observing DHI-sourced queries documentation ^@^	
Never compile as documentation	6 (30.0)
Documentation made but not available on-hand	6 (30.0)
Documentation misplace	4 (20.0)
Access to documentation denied	4 (20.0)

@ – Attitude/perception questions, β – Practice questions

In order to keep track of data submission, a variety of approaches were used, ranging from 57.6% that countersigned against the physical reports to 18.2% relying on DHIS-2 dashboards for tracking. Notably however, one participant did not perform any tracking of their data entry.

An overwhelming majority (93.9%) of participants indicated that they received data queries from DHI, concerning the data they submit (Table 3). Among these, 54.8% received data queries by email, while 35.5% received queries through more than one channels, mail plus SMS/WhatsApp being the predominant combination. Whilst very few did not receive queries from DHI, only 42.4% of participants who received queries from DHI were able to provide documentation of the queries that they had received and/or had to address in the three months prior to the survey. Reasons provided by participants that were unable to provide documentation for queries received, ranged from documentation kept but not being available on-hand (31.6%) to denied access to the documentation (21.1%). Of the 1556 health facilities that routinely submit reports within the 14/34 participants that provided documentation of DHI-sourced queries, 18.2% were implicated in the queries observed, having to address an arithmetic mean of 4.2 queries per facility.

On keeping track of the queries received by DHM from DHI, participants primarily referred to the original message in the format they had received it. Particularly, they referred to: electronic messages on SMS/WhatsApp (61.3%) and email (29.0%); documented queries on paper (19.4%); or on self-summarized phone conversation, written on paper (16.1%), among others (Table 3).

### Data analysis and use

Concerning analysis and use of data compiled within DHM offices, majority of participants (52.9%) analyzed their data on a monthly basis, over the past 12 months (Table 4). From these analyses, a variety of outputs were generated, including mainly summary tables, endemic channel plots [26] commonly known in Uganda as normal channel plots, trend plots, pie charts, and, in nearly a third of the cases (29.4%), as a combination of all four outputs.

**Table 4:** Attitude and practices towards analysis and use of routinely reported data, among district HMIS managers

Question	n (%) N = 34
Times analyzed data internally, over past 12 months, for report(s) generated ^β^	
Zero—Twice	5 (14.7)
Quarterly	11 (32.4)
Monthly	18 (52.9)
Other	1 (2.9)
Kind of results normally generated from internal analyses ^β^	
Summary Tables	32 (94.1)
Normal Channel plots	22 (64.7)
Trend plots	20 (58.8)
Pie Charts	19 (55.9)
Bar/Line graphs	4 (11.8)
Maps	1 (2.9)
Times received external analytical feedback generated from your data, over past 12 months ^β^	
Zero-Twice	12 (35.3)
Quarterly	12 (35.3)
Monthly	4 (11.8)
Other	6 (17.6)
Kind of externally generated analytical feedback mainly received by participants ^β^	
Plain acknowledgement of receipt of submitted data	11 (32.4)
Queries on data submitted	19 (55.9)
Data quality performance assessment	23 (67.6)
Other	2 (5.9)
Kind of results/outputs you like to be included in external feedback ^@^	
Summary tables	29 (85.3)
Trend plots	24 (70.6)
Normal channel plots	20 (58.8)
Pie charts	18 (52.9)
Maps	9 (26.5)
Desired regularity to conduct analyses on routinely generated/collected data ^@^	
Weekly	6 (17.6)
Monthly	11 (32.4)
Quarterly	14 (41.2)
Other	3 (8.8)

@ – Attitude/perception questions, β – Practice questions

With the exceptions of desired regularity to conduct analyses and time that external analytical feedback was received, participants used more than one of the available options for all responses here. For instance, one participant analyzed data internally, both on a monthly and quarterly bases

On the other hand, participants received from sources external to their DHM offices, analytical outputs from the data they generated or submitted. Over the past 12 months, 67.6% received feedback quarterly or less frequently, excluding no feedback at all (Table 4). The rest (23.5%) received external feedback on a monthly or more frequent basis. This externally generated analytical feedback was mainly in the form of either data quality performance assessments, queries to resolve, acknowledgement of receipt of the data submitted, or a combination of these, among others.

Assessment of the kinds of analytical outputs that would be desirable to receive from any external sources, showed that summary tables, trend, or endemic channel plots were prioritized at 85.3, 70.6, or 58.8%, respectively. Notably, maps were mentioned as desired feedback, by 26.5% of participants, a much higher proportion than actively generated maps. However, a fairly comparable proportion desired to receive pie charts (52.9%) as actively generated their own (55.9%). From within DHM offices, a desired analysis regularity by participants ranged between 17.6% (weekly) and 41.2% (quarterly).

**Fig. 2:**
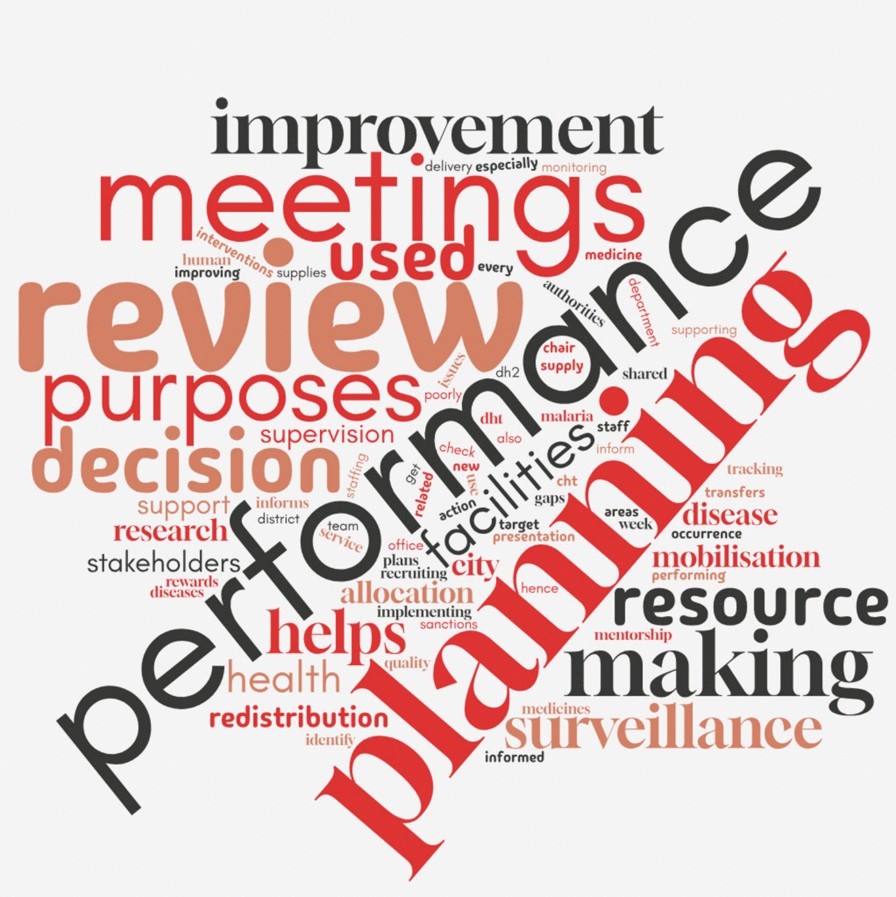
Key-words from reported uses of results from internal analysis of health facility data, by district HMIS managers

The study did not assume standardized uses of analytical outputs from routine data, particularly the internally generated outputs by and for the DHM. Uses of these outputs, explored using an open-ended question, were summarized using key-words (Fig. 2). The top ten notable key words associated with uses of routine data or its analytical outputs by and for the DHM were; performance, planning, review, meetings, purposes, decision, making, improvement, resource, and facilities. Notable uses associated with these key words included use of this data for: performance review meetings, planning purposes, decision-making, resource allocation, performance of or supporting facilities, and identifying areas for improvement.

On the other hand, a subjective selection of ten key words with the lowest occurrence included, staffing, recruiting, rewards, sanctions, transfers, redistribution, gaps, action, supervision, and mentorship (Fig. 2). Associated notable uses of routine data analytical outputs included, use of this data: to inform staffing, recruiting staff, when to get new staff or transfer others; for rewards and sanctions, for supplies redistribution, to identify gaps, for action improvement, in support supervision, and for targeted mentorship.

### Support supervision on HMIS related matters

*DHI support supervision of DHM for HMIS management*: A considerable proportion of participants (47.1%) had not received any DHI support supervision over the 12 months prior to the survey (Table 5). Nevertheless, 41.2% had received one to two DHI support supervision visits. Over the shorter duration of 6 months prior to the survey, the proportion of participants that had not received any support supervision increased by 11.7 percentage points, with those who had received a single supervision visit more than doubling to 32.4%. Assessing for district-level managers’ preferred frequency of DHI support supervision visits, 70.6% of participants cited quarterly, followed by 20.6% choosing a once every six months preference. Assessing for possession of a support supervision report where visiting supervisors record key findings and recommendations, 84.4% had no report due primarily to non-use of this HMIS tool. Among the very few observed reports, the latest visit had happened more than twelve months prior to the survey.

**Table 5:** Attitude and practices towards routine surveillance support supervision, among district HMIS managers

Question	n (%) N = 34
Can we have a look at your support supervision report book? ^β^	
Yes—observed	5 (15.6)
No—Not observed	27 (84.4)
Reasons why support supervision book was not observed ^β^	
Not in use / office doesn’t have one	23 (85.2)
Not accessible now / Misplace	3 (11.1)
Not yet printed a new one	1 (3.7)
Number of times you received support supervision from DHI in the past 12 months ^β^	
Once	5 (14.7)
Twice	9 (26.5)
Quarterly	2 (5.9)
Other	2 (5.9)
None	16 (47.1)
Number of times you received support supervision from DHI in the past 6 months ^β^	
Once	11 (32.4)
Twice	2 (5.9)
Other	1 (2.9)
None	20 (58.8)
Preferred number of DHI support supervision visits in a year ^@^	
Once	1 (2.9)
Twice	7 (20.6)
Monthly	2 (5.9)
Quarterly	24 (70.6)
Times you provided HMIS-related support supervision to your health facilities in past 12 months ^β^	
1–3 times	7 (20.6)
4–6 times	19 (55.9)
Monthly	6 (17.6)
Other	2 (5.9)
Times you provided HMIS-related support supervision to your health facilities in past 6 months ^β^	
1–3 times	22 (64.7)
4–6 times	8 (23.5)
None	3 (8.8)
Other	1 (2.9)
Times you would like to provide HMIS-related support supervision to your facilities in 12 months ^@^	
Monthly	11 (32.4)
Quarterly	23 (67.7)

@ – Attitude/perception questions, β – Practice questions

*District HMIS management’s support supervision of their health facilities on data*: A majority (55.9%) of participants indicated that they had conducted supervision four times or quarterly, over the 12 months before the survey, making the four to six months group the best performing, followed by the one to three times group at 20.6% (Table 5). Considering a shorter assessment duration of the past six months prior to the survey, majority of the participants (64.7%) had conducted support supervision for between one to three times in their health facilities, heavily influenced by those who had supervised twice over those last six months.

When asked about their preferred frequency of providing HMIS-related support supervision to health facilities, 67.7% of participants preferred quarterly while the rest monthly timescales. Among reasons cited for the quarterly timescale preference, participants indicated that this helped them align this activity with district budgeting, resource availability and reporting timelines, which are quarterly, in each case.

**Fig. 3:**
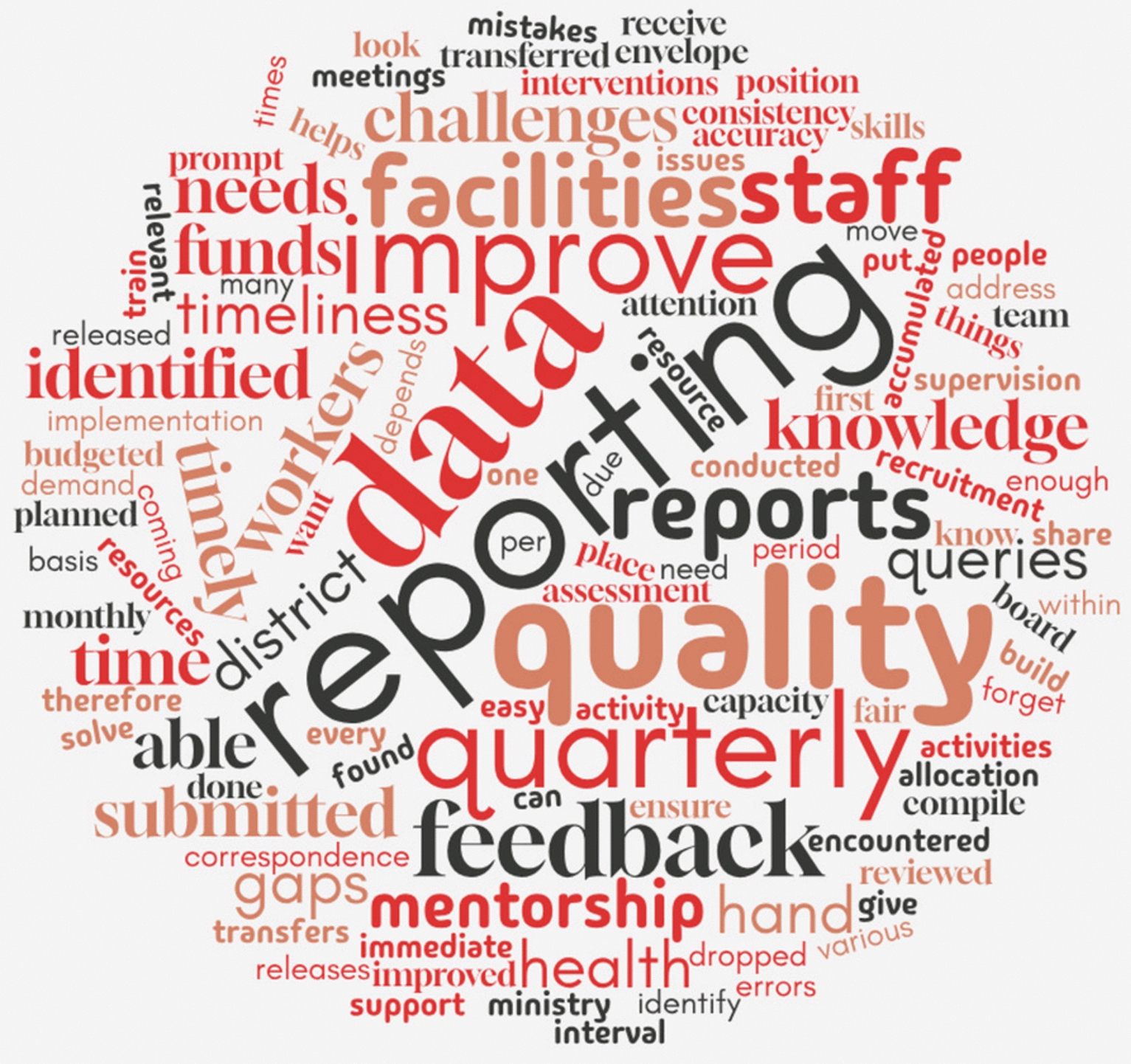
Key-words from reasons for monthly and/or quarterly supervision of health facilities, by district HMIS managers

An assessment of reasons why participants preferred the frequencies of quarterly or monthly timescales for support supervision of their health facilities highlighted key terms. The top ten key-words included reporting, data, quality, improve, quarterly, facilities, feedback, staff, challenges, and funds (Fig. 3). Important reasons associated with these key words included: To improve quality of reporting; the many data queries received needing immediate attention; to align with funds or resources’ availability and reports being due on a quarterly timescale, though data is received on monthly timescale; To ensure prompt feedback to implementation teams, the facility members; To address skills among recruits, given high staff turn-over; and, to address reporting challenges in a timely way. Other notable key-words at the lowest densities included transfers, skills, look, fair, enough, attention, and hand. The reasons associated with these key words included: Frequent staff transfers being disruptive on available skills; enabling in-depth look at reports submitted; considering fair enough time for adjustments; and ensuring first hand attention to challenges.

## Discussion

### Primary findings

This national cross-sectional study found that a well-educated, albeit male-dominated, cadre of fairly young managers was in charge of routine HMIS reporting within district-level health offices across endemicity regions of Uganda. This critical human resource was characterized by highly capable individuals handling the vital tasks pertaining to surveillance, given the necessary resources and/or support. As such, there were good reasons for confidence in the human resource administering routine surveillance to produce valid and high-quality data for malaria and other reportable diseases’ control or elimination.

Results showed that more than one person at each district-level health office was actively engaged in routine HMIS reporting activities, including regularly reviewing, for quality, the data reports submitted to the district. Moreover, district-level managers were able to enlist the support of facility-level data staff whenever necessary, a clear indication of wide-spread collaboration on data and routine surveillance within the districts. At this performance level, however, up to a quarter of managers interviewed did not document the issues, on quality or accuracy, identified in the data reports received from their health facilities when reviewed at their district-level health offices. Whilst majority of non-documenters of data issues opted for the more pragmatic approach of calling the corresponding facility staff for clarifications, this practice, if inconclusive at the time of identifying an issue, increases the chances of issues remaining perpetually unresolved with little to no likelihood of follow-up. This may subsequently propagate data errors or missingness to next reporting levels, affecting overall data quality. Furthermore, with less than one third of the queries documenters able to easily access their queries record, the impetus to address these queries may need further strengthening through say, a standardized procedure of documenting and resolving identified data issues. Overall, a lack of defined standards and/or procedures to document, track, and monitor data quality and thereby mitigate data issues was evident.

The surveillance system in Uganda boasts of high health facility reporting rates, corroborated by this study [27]. However, results show that up to a quarter of the realized reporting depends on extensive follow-up with reporting health facilities by district-level managers. This highlights a demand for resources, such as time and communication costs, to perform this follow-up, but also that interventions to improve voluntary timely submission at the data sources may be vital. Importantly also, there need to be mutually beneficial feedback loops for DHM and other levels–not simply to correct mistakes, but also to the impact of the submitted data.

Relatedly, a majority of district-level managers completing their data entry into DHIS-2 within the first 15 days of the new month directly reflects the time during which all follow-up for data submission was ensured. This suggests that the first half of the new month is a rather engaging time for district-level HMIS managers to ensure full surveillance compliance and therefore, may not be suitable for other activities, such as those requiring these managers’ involvement outside of their duty stations.

Following data submission, an overwhelming majority of district-level managers received queries from the highest level, DHI, ostensibly due to a combination of vigilance at the higher level and laudable functionality of the data management system at auto-generation of queries. However, there was low use of queries’ documentation, with much less than half of participants able to find or show their queries record. This amplifies the urgency for heightened vigilance with queries-follow-through by these district-level managers, given a fairly high average query rate of four per facility. Such vigilance may lead to further improvement of data quality, if intentionally maintained.

For internal use, an encouraging majority of district-level managers (nearly 70%) conducted analysis of their reported data monthly, a higher analysis regularity than the quarterly desired by a slight majority. The internally generated analytical output formats were closely mirrored by desired analytical output formats, be they externally sourced. A notable difference was in the quest for geographical outputs at 27% versus nearly absent practice to generate them. These findings suggest a good mix of district-level managers’ awareness, skills, and confidence around the use of summary tables, endemic channel plots, trend plots, and pie charts, as well as their need for advanced skills in generating the same. Moreover, they may indicate widespread demand for analytical feedback that incorporates interpretation-friendly results formats like pie charts or the country-widely understood and promoted endemic channel plots. These plots, comprising time series of the monthly median and the upper 3rd quartile of case counts, are used to observe time points where monthly counts exceed the 3rd quartile—indicating an epidemic—and are recommended by WHO for epidemic monitoring [28]. Overwhelmingly, however, the difference between nearly absent use of maps (3%) as an internally generated analytical output and desire for maps (26.5%) to be included in externally sourced analytical outputs was profound. This may be suggestive of a skills gap in generating maps for surveillance data interpretation, given low use of maps generated internally, coupled with a good understanding of their utility, given the high desire for maps generated externally, among district-level managers.

Whilst, the different analytical outputs, internally generated or desired to be outsourced, weren’t each linked to intended specific application(s) or purpose(s), an open-ended discussion of general uses of these outputs raised vital insights. The most cited, and therefore, most cross-cutting uses of surveillance analytical outputs, included: general and health facility performance review; planning, decision-making, and resource allocation; as well as, identification of gaps or areas for improvement and supporting health facilities. This broad array of widely recognized uses of routine data analytical outputs across districts suggests a high level of regard and utility of these routine data by HMIS managers across Uganda. This could be supportive evidence towards achieving the data-use strategic objective of the prevailing malaria strategic plan 2021–2025, at the districts level [15]. Among the notable but little mentioned data-uses, however, were: supplies re-distribution, that refers to situations where some facilities find themselves overstocked with a particular commodity, at a time when other(s) are stocked out, and the overstocked share(s) with those stocked out; targeted mentorship that may point to human resource capacity building, particularly given that problem of high staff turnover was raised by several participants; and, support supervision. Unfortunately, the very low consideration of data to inform support supervision correlates with the apparent linking of support supervision primarily to resource availability rather than identified gaps, which could be an area for systemic improvement.

To ensure full adherence to the required data reporting standards at any given level, support supervision, from the next higher level internal or otherwise, is extremely critical [29, 30]. Support supervision enables problem-root-cause identification concurrently with collective solution development at the highest proximity to the source [31]. Whilst DHI support supervision of the district-level managers is happening, there are gaps between desired (district) and actual (DHI) regularity of this supervision as well as in documentation of findings or recommendations when visits happen. Districts largely felt that a visit once every quarter from the DHI would be preferred to ensure good performance. However, a lot still needs to be done since approximately half of district-level managers reported having not received a single supervision visit through the past year and an even greater majority had no tools to record findings or recommendations of such a visit.

Regarding district-level managers’ provision of support supervision to their facilities, a slightly smaller difference was observed between their current practice and their desired frequency. This closeness of perception and practice performance could arguably have been subject to desirability bias. However, with the main reason for the quarterly timescale for majority in both the desired and practiced time-scale, being ensured alignment with resource availability and reporting timelines, it’s unlikely that desirability significantly influenced participants’ responses. Nevertheless, the apparent widely entrenched idea of linking supervision activities to resource-available cycles may risk establishing seasonal vigilance across the reporting system rather than intentionally sustained efforts to improve evidence generation and health service provision.

### Strengths and limitations of the study

Notable merits of this study include, firstly this comprised a broad cross-section of the country, including both rural and urban districts, as well as fully-fledged cities of Uganda. Moreover, the districts were selected with careful consideration of the diverse epidemiological profile of Uganda classified into 15 malaria endemicity regions with nearly 100% response rate. Secondly, the timing of the survey strategically fell well within the prevailing malaria reduction and eliminations strategic duration spanning 2021–2025 at which point, all intended strategic objectives would be in advanced phases [15]. This therefore, contributes to a broader performance review of routine surveillance.

Merits of this study notwithstanding, there were limitations worthy of mention. First, whilst several open-ended questions were included in this survey, a fully comprehensive qualitative explanation or interpretation of the observed perceptions and practices may not be possible, limiting potential insights drawn. However, numerous questions provided complementary findings to each other and together with the open-ended discussion results, these ensured consolidated learnings from this study. Secondly, most studies assessing perceptions and practices often consider quantitative performance on these. However, this was not feasible in this study due to the small number of district-level offices and thereby, participants. Nonetheless, such quantitative estimates predominantly inform knowledge performance, which was not a practical focus of this study. Thirdly, the small numbers further limited capacity to assess and infer any potential associations between observed perceptions or practices and potential factors, albeit without detriment to the credence of the main study findings. Lastly, whilst mistakes in the data were a key part of this assessment, the actual mistakes identified by DHM officials were not particularly examined to assess their extent or classify them so as to identify patterns and therewith infer possible solutions.

## Conclusion

More than one individual manages district-level routine reporting for malaria, with engagement of health facility officials common, where needed, and the first 15 days of the new month a critical window to ensure complete data reporting. Analytical outputs from routine data, generated by participants remain heavily monitoring-focused and very limited towards impact assessment suggesting a skills gap or underutilization of routine data. The current strong linking of support supervision to resource availability cycles limits its capacity to shore up data quality and general health system improvement. These findings point to the need for districts to establish and/or ensure they adhere to standard operating procedures for data reporting, follow-up, documentation, tracking, and feedback mechanisms; improve health system-wide appreciation and adoption of support supervision; as well as institutionalize advanced analytical skills training aligned with key routine data use-cases to ensure cutting-edge evidence, improved data use, and, thereby, impactful decision-making.

## Supplementary Information

Additional file 1.

## Data Availability

Data can be shared by the corresponding author on reasonable request.

## Abbreviations

CSV: Comma separated version
DHI: Division of Health Information
DHIS-2: District Health Information Software–Version 2
DHM: District or City Health Management Officials
HMIS: Health Management Information System
PfP: Private for profit
SMS: Short Message Service
