# Supplementary material for: Attitude and practices of district-level HMIS managers towards malaria routine reporting in Uganda: a 2024 cross-sectional study
Source: Malar J. 2026 Jun 1;25:281. doi: 10.1186/s12936-026-05951-8 (PMC13440161; doi:10.1186/s12936-026-05951-8)
Supplement: Supplementary file 1 [file 12936_2026_5951_MOESM1_ESM.docx]

Table 1. District HMIS manager attitude and practices survey participants’ demographics summary

| **Variable** | **n (%)**  N=34 |
| --- | --- |
| **Gender** | |
| Male | 20 (58.8) |
| Female | 14 (41.2) |
| **Age** | |
| <=30 years | 7 (20.6) |
| 31 - 45 years | 22 (64.7) |
| 46 – 60 years | 5 (14.7) |
| **Highest level of education** | |
| Certificate | 1 (2.9) |
| Diploma | 9 (26.5) |
| Degree | 11 (32.4) |
| Post-graduate | 13 (38.2) |
| **Participant Cadre** | |
| HMIS focal person | 8 (23.5) |
| Biostatistician | 24 (70.6) |
| Other | 2 (5.9) |
| **Number of health facilities reporting on malaria to the district office –** *(min, median, max)* | |
| Minimum | 6 |
| Median | 28 |
| Maximum | 1043 |

**Figure 1. Study sites of districts and cities across Uganda, enrolled into the survey**

Table 2. Attitude and practices towards routine data recording and review, among district HMIS managers

| **Question** | **n (%)**  N=34 |
| --- | --- |
| **How do you keep track of facilities that have/have not submitted a monthly report? ^β^** | |
| Use electronic spreadsheet | 25 (73.5) |
| Use paper spreadsheet / register | 11 (32.4) |
| Monitor on DHIS-2 or mTrac | 7 (20.6) |
| **When do you consider a monthly report delayed? ^@^** | |
| >7 days into the new month | 23 (67.6) |
| 2-10 days into the new month | 6 (17.7) |
| >=11 days into the new month | 5 (14.7) |
| **When do you get in touch with health facility if monthly report is delayed? ^β^** | |
| 5-10 days into the new month | 21 (61.8) |
| 10-15 days into the new month | 12 (35.3) |
| >15days into the new month | 1 (2.9) |
| **Do you document mistakes found in monthly reports you receive? ^β^** | |
| Yes | 26 (76.5) |
| No | 8 (23.5) |
| **How do you communicate mistakes identified in monthly reports to health facility? ^β^** | |
| Phone call | 30 (88.2) |
| SMS/WhatsApp message | 16 (47.1) |
| Send documented query on paper | 3 (8.8) |

@ – Attitude/perception questions, β – Practice questions

*For both keeping track of facilities submitting reports and communicating mistakes identified in the reports, some participants used more than one option.*

Table 3. Attitude and practices towards routine data reporting, among district HMIS managers

| **Question** | **n (%)**  N=34 |
| --- | --- |
| **When do you enter the reports data into DHIS-2? ^β^** | |
| Daily | 3 (8.8) |
| In the first week of the new month | 6 (17.6) |
| By the 15^th^ day of the new month | 20 (58.8) |
| Other | 5 (14.7) |
| **How do you keep track of reports that you have submitted in DHIS-2? ^β^** | |
| Counter sign on reports | 19 (55.9) |
| Keep a data entry ledger | 13 (38.2) |
| Rely on DHIS-2 dashboards | 7 (20.6) |
| Not keeping track | 1 (2.9) |
| **Do you receive queries from DHI concerning what you submitted? ^β^** | |
| Yes | 32 (94.1) |
| No | 2 (5.9) |
| **How are data queries from DHI communicated to you? ^β^** | |
| Email | 18 (52.9) |
| SMS/WhatsApp electronic message | 14 (41.2) |
| Phone call | 8 (23.5) |
| Documented on paper | 3 (8.8) |
| **How do you keep track of the queries received from DHI? ^β^** | |
| Refer to SMS/WhatsApp message received | 19 (55.9) |
| Refer to email received | 9 (26.5) |
| Refer to documented query on paper | 7 (20.6) |
| Refer to my written summary from phone conversation | 5 (14.7) |
| other | 5 (14.7) |
| **Was documentation of DHI-sourced queries availed to study staff for observation? ^β^** | |
| Yes | 14 (41.2) |
| No | 20 (58.8) |
| **Summary of observed DHI-sourced queries documentation** | |
| Number of queries documented over 3 months | 1201 |
| Health facilities implicated in queries recorded over 3 months | 283 |
| **Reasons for not observing DHI-sourced queries documentation ^@^** | |
| Never compile as documentation | 6 (30.0) |
| Documentation made but not available on-hand | 6 (30.0) |
| Documentation misplace | 4 (20.0) |
| Access to documentation denied | 4 (20.0) |

@ – Attitude/perception questions, β – Practice questions

Table 4. Attitude and practices towards analysis and use of routinely reported data, among district HMIS managers

| **Question** | **n (%)**  N=34 |
| --- | --- |
| **Times analyzed data internally, over past 12 months, for report(s) generated ^β^** | |
| Zero - Twice | 5 (14.7) |
| Quarterly | 11 (32.4) |
| Monthly | 18 (52.9) |
| Other | 1 (2.9) |
| **Kind of results normally generated from internal analyses ^β^** | |
| Summary Tables | 32 (94.1) |
| Normal Channel plots | 22 (64.7) |
| Trend plots | 20 (58.8) |
| Pie Charts | 19 (55.9) |
| Bar/Line graphs | 4 (11.8) |
| Maps | 1 (2.9) |
| **Times received external analytical feedback generated from your data, over past 12 months ^β^** | |
| Zero-Twice | 12 (35.3) |
| Quarterly | 12 (35.3) |
| Monthly | 4 (11.8) |
| Other | 6 (17.6) |
| **Kind of externally generated analytical feedback mainly received by participants ^β^** | |
| Plain acknowledgement of receipt of submitted data | 11 (32.4) |
| Queries on data submitted | 19 (55.9) |
| Data quality performance assessment | 23 (67.6) |
| Other | 2 (5.9) |
| **Kind of results/outputs you like to be included in external feedback ^@^** | |
| Summary tables | 29 (85.3) |
| Trend plots | 24 (70.6) |
| Normal channel plots | 20 (58.8) |
| Pie charts | 18 (52.9) |
| Maps | 9 (26.5) |
| **Desired regularity to conduct analyses on routinely generated/collected data ^@^** | |
| Weekly | 6 (17.6) |
| Monthly | 11 (32.4) |
| Quarterly | 14 (41.2) |
| Other | 3 (8.8) |

@ – Attitude/perception questions, β – Practice questions

*With the exceptions of desired regularity to conduct analyses and time that external analytical feedback was received, participants used more than one of the available options for all responses here. For instance, one participant analyzed data internally, both on a monthly and quarterly bases.*

Table 5. Attitude and practices towards routine surveillance support supervision, among district HMIS managers

| **Question** | **n (%)**  N=34 | | | |
| --- | --- | --- | --- | --- |
| **Can we have a look at your support supervision report book? ^β^** | | | |  |
| Yes – observed | | | 5 (15.6) | |
| No – Not observed | | | 27 (84.4) | |
| **Reasons why support supervision book was not observed ^β^** | | | | |
| Not in use / office doesn’t have one | | 23 (85.2) | | |
| Not accessible now / Misplace | | 3 (11.1) | | |
| Not yet printed a new one | | 1 (3.7) | | |
| **Number of times you received support supervision from DHI in the past 12 months ^β^** | | | | |
| Once | 5 (14.7) | | | |
| Twice | 9 (26.5) | | | |
| Quarterly | 2 (5.9) | | | |
| Other | 2 (5.9) | | | |
| None | 16 (47.1) | | | |
| **Number of times you received support supervision from DHI in the past 6 months ^β^** | | | | |
| Once | 11 (32.4) | | | |
| Twice | 2 (5.9) | | | |
| Other | 1 (2.9) | | | |
| None | 20 (58.8) | | | |
| **Preferred number of DHI support supervision visits in a year ^@^** | | | | |
| Once | 1 (2.9) | | | |
| Twice | 7 (20.6) | | | |
| Monthly | 2 (5.9) | | | |
| Quarterly | 24 (70.6) | | | |
| **Times you provided HMIS-related support supervision to your health facilities in past 12 months ^β^** | | | | |
| 1-3 times | 7 (20.6) | | | |
| 4-6 times | 19 (55.9) | | | |
| Monthly | 6 (17.6) | | | |
| Other | 2 (5.9) | | | |
| **Times you provided HMIS-related support supervision to your health facilities in past 6 months ^β^** | | | | |
| 1-3 times | 22 (64.7) | | | |
| 4-6 times | 8 (23.5) | | | |
| None | 3 (8.8) | | | |
| Other | 1 (2.9) | | | |
| **Times you would like to provide HMIS-related support supervision to your facilities in 12 months ^@^** | | | | |
| Monthly | 11 (32.4) | | | |
| Quarterly | 23 (67.7) | | | |

@ – Attitude/perception questions, β – Practice questions

Figure 2. Key-words from reported uses of results from internal analysis of health facility data, by district HMIS managers

Figure 3. Key-words from reasons for monthly and/or quarterly supervision of health facilities, by district HMIS managers
